# Complete Genome Sequence of Salmonella enterica Serovar Newport Myophage Melville

**DOI:** 10.1128/MRA.00255-19

**Published:** 2019-04-25

**Authors:** Kailun Zhang, Yicheng Xie, Chandler J. O'Leary, Mei Liu, Jason J. Gill

**Affiliations:** a Center for Phage Technology, Texas A&M University, College Station, Texas, USA; Loyola University Chicago

## Abstract

Multiple antimicrobial-resistant strains of Salmonella enterica serovar Newport have been recorded. Study on phages infecting *S.* Newport may provide new therapeutics or diagnostics for this pathogen. Here, we describe the complete genome sequence of the T4-like phage Melville that uses *S.* Newport as one of its hosts.

## Abstract (precis)

Multiple antimicrobial-resistant strains of Salmonella enterica serovar Newport have been recorded. Study on phages infecting *S.* Newport may provide new therapeutics or diagnostics for this pathogen.

## ANNOUNCEMENT

The CDC listed Salmonella enterica serovar Newport as one of the top three *Salmonella* serotypes associated with human infections (1) and foodborne outbreaks (2, 3) in the United States. However, several strains of *S.* Newport display resistance to multiple classes of antimicrobials, including expanded-spectrum cephalosporins (4–6). The study of *S.* Newport phages will provide insights into the control of *Salmonella* bacteria.

Myophage Melville was isolated from a mixed wastewater sample from Austin, TX in August 2016 using *S*. Newport as the host. Host bacteria were cultured on tryptic soy broth or agar (Difco) at 37°C with aeration. The phage was isolated and propagated by the soft agar overlay method (7). Phage genomic DNA was prepared using a modified Promega Wizard DNA cleanup kit protocol (8). Pooled indexed DNA libraries were prepared using the Illumina TruSeq nano low-throughput (LT) kit, and the sequence was obtained from the Illumina MiSeq platform using the MiSeq v2 500-cycle reagent kit, following the manufacturer’s instructions, producing 667,982 paired-end reads for the index containing the phage genome. Quality-controlled (FastQC; http://www.bioinformatics.babraham.ac.uk/projects/fastqc/) trimmed (FASTX-Toolkit 0.11.6; http://hannonlab.cshl.edu/fastx_toolkit/) reads were assembled using SPAdes 3.5.0 (9) into a contig at 132.8-fold coverage. The genome sequence was completed by PCR using primers (5′-TCTTCATAGCATGGGCACATATC-3′ and 5′-GGCGGGTGGTTTGAAGTAA-3′) facing off the ends of the assembled contig and Sanger sequencing of the resulting product, with the contig sequence manually corrected to match the resulting Sanger sequencing read. Protein-coding genes were predicted by Glimmer 3.0 (10) and MetaGeneAnnotator 1.0 (11), with manual correction. tRNA genes were analyzed using ARAGORN 2.36 (12). Protein functions were predicted based on sequence homology by BLASTp 2.2.28 (13). Conserved domain searches were conducted in InterProScan 5.15-5.40 (14). All analyses were conducted at default settings via the Center for Phage Technology (CPT) Galaxy (15) and WebApollo (16) interfaces (https://cpt.tamu.edu/).

The Melville genome (159,323 bp) has a G+C content of 37%, a level lower than that of *Salmonella* spp. (∼50%) (17). Genes encoding dCMP hydroxymethylase that produce hydroxymethyl cytosine (HMC) DNA were identified, suggesting that there is protection from nucleases that degrade host DNA. Genes for holin (class III with a predicted single transmembrane domain [TMD] in an N-in, C-out topology), endolysin (murein hydrolase), and spanins for lysis of the host are distributed in the Melville genome. There is one self-splicing group I intron in the gene encoding thymidylate synthetase. The presence of the inner membrane protein *imm* (immunity) gene in Melville indicates the ability to exclude superinfecting phage.

Melville is a T4-like phage and belongs to the genus *S16virus*. Melville encodes homologs of the protector from prophage-induced early lysis genes *rIIA* and *rIIB*, as is common among T4-like phages. It shares 93.9% and 90.5% whole-genome DNA sequence identity by BLASTn with the *Salmonella* phage STML-198 (GenBank accession numbers NC_027344) and *Salmonella* phage vB_SenMS16 (S16; GenBank accession number NC_020416), respectively. As is the case with phage S16, Melville contains a tandem gene duplication of the predicted capsid vertex protein (GenBank accession numbers ATN93139 and ATN93140). The long tail fiber distal subunit of Melville (GenBank accession number ATN93217) has 76% identity with that of phage S16, which recognizes the outer membrane protein OmpC and has an unusually broad host range within the genus *Salmonella* (18).

### Data availability.

The genome sequence of phage Melville was deposited under GenBank accession number MF957259. The associated BioProject, SRA, and BioSample accession numbers are PRJNA222858, SRR8788210, and SAMN11259695, respectively.
